# Proportion of depression and associated factors among HIV-positive youth attending antiretroviral therapy clinics at public hospitals in Gamo and Ari zones, South Ethiopia: A facility-based cross-sectional study

**DOI:** 10.1371/journal.pone.0337588

**Published:** 2025-12-05

**Authors:** Temesgen Mohammed Toma, Abraham Anbesie Sapo, Habtamu Wana Wada, Kassahun Tamene Andarige, Rahel Abera Alula, Fasika Merid Malimo, Habtamu Samuel Goda, Mintesinot Melka Gujo, Agune Ashole Alto, Tamirat Gezahegn Guyo

**Affiliations:** 1 Public Health Emergency Management Directorate, South Ethiopia Region Health Bureau Public Health Institute, Jinka, Ethiopia; 2 Department of Public Health, Arba Minch College of Health Sciences, Arba Minch, Ethiopia; 3 Department of Environmental Health, Arba Minch College of Health Sciences, Arba Minch, Ethiopia; 4 Department of Midwifery, Arba Minch College of Health Sciences, Arba Minch, Ethiopia; 5 Deputy Director General, South Ethiopia Region Health Bureau Public Health Institute, Jinka, Ethiopia; 6 Director General, South Ethiopia Region Health Bureau, Public Health Institute, Jinka, Ethiopia; 7 Regional Data Management Center, South Ethiopia Region Health Bureau Public Health Institute, Jinka, Ethiopia; PLOS: Public Library of Science, UNITED KINGDOM OF GREAT BRITAIN AND NORTHERN IRELAND

## Abstract

**Background:**

Even though the co-occurrence of Human Immune-Deficiency Virus/Acquired Immune-Deficiency Syndrome (HIV/AIDS) and depression is the main cause of morbidity and mortality in youth living with HIV/AIDS, in Ethiopia, there is a scarcity of evidence on depression and associated factors. Hence, this study aimed at the assessment of depression and associated factors among HIV-positive youth attending antiretroviral therapy (ART) clinics at public hospitals in Gamo and Ari Zones, South Ethiopia.

**Method:**

A facility-based cross-sectional study was conducted from March 1, 2024, to April 30, 2024, among HIV-positive youth attending ART clinics at public hospitals in Gamo and Ari Zones. A systematic random sampling technique was used to select 343 study participants. Descriptive statistics were used to describe variables. Binary logistic regression was used to identify factors associated with depression. Variables with p-value <0.25 on the bivariable logistic regression analysis were candidates for the multivariable logistic regression analysis. Adjusted odds ratio (AOR) with a corresponding 95% confidence interval (CI) was used to determine the strength of association. A p-value <0.05 was used to set a statistical significance.

**Result:**

A total of 343 HIV-positive youths were included in the study, with a 96.4% response rate. The proportion of depression was 22.2% (95%CI: 18.1%, 26.9%). Poor psychosocial support (AOR = 2.87; 95%CI: 1.18, 6.98), death of parents (AOR = 3.28; 95%CI: 1.73, 6.20), substance use (AOR = 3.44; 95%CI: 1.60, 7.43), advanced WHO clinical staging (AOR = 4.35; 95% CI: 1.97, 9.61), and initiation on a non-Dolutegravir (DTG)-based regimen (AOR = 2.72; 95%CI: 1.15, 6.42) were associated with depression among HIV positive youths.

**Conclusion:**

Depression is found to be a significant public health problem in the study settings. Poor psychosocial support, death of parents, substance use, advanced WHO clinical staging, and initiation on a non-DTG-based regimen were significant predictors of depression. Special attention should be given to those with poor psychosocial support, substance use, orphaned, and poor baseline clinical characteristics. Moreover, early identification and treatment of youths with depression during routine HIV care is essential to avert depression.

## Introduction

Depressive disorder (also known as depression) is a common mental disorder with a variety of emotional, physical, and behavioral symptoms characterized by poor concentration, feelings of excessive guilt or low self-worth, thoughts about dying or suicide, disrupted sleep, and decreased energy for 14 days or longer [1,2]. It can be classified as mild, moderate, or severe based on the frequency and severity of symptoms, and the effect on the person’s functioning [2]. Depression in HIV-positive patients results from a complex interaction of social, psychological, and biological factors [3]. Despite a strong linkage between depression and HIV/AIDS, depression is a distinct disorder that can be diagnosed and treated while a person is receiving treatment for HIV/AIDS [4].

According to the World Health Organization (WHO), youths are persons aged 15 − 24 years [5]. Youth represent about 15% of the global population but account for an estimated 28% of new HIV infections [6]. HIV-positive youth face multiple mental health problems, specifically depression, on top of bearing and coping with the developmental phase challenges [7]. Markedly, HIV-positive youths have greater rates of depression than the overall population and as well as higher rates than HIV-negative youth and youth with other life-threatening diseases [8].

Depression is the leading cause of ill health and disability globally [9]. In the circumstance of HIV/AIDS, depression is an often overlooked but potentially risky disorder that can affect not only the quality of life, work, relationships, and adherence to therapeutic care but also survival [10]. Depression is prevalent among youths living with HIV [11]. More than 25% of HIV-positive youth reported that they are suffering from depression globally [12]. Depression among youths living with HIV (YLWH) serves as a risk factor for non-adherence, low viral suppression, risky sexual behaviors, faster progression to AIDS, and earlier death [6]. The consequence of depression in YLWH also continues into adulthood; wherein it is associated with poor quality of life and increased risk of HIV/AIDS transmission to others [13].

Depression can lead to thoughts of suicide and dying, with over 700,000 people dying due to suicide every year. Suicide is the fourth leading cause of death in 15–29-year-olds [2]. Depression is identified as a major mental health problem of YLWH globally. However, HIV/AIDS alone is becoming the disease of youth, and as a result, globally the second and in Africa the leading cause of mortality among youth [14]. The number of individuals with depression among youth globally is going up [1]. Different studies conducted among YLWH showed that the raised magnitude of depression in various parts of the world ranges from 27.8% − 84.4% [7,15–18]. In Africa, the prevalence of depression among YLWH ranges from 27% to 52.6% [19–23]. In low and middle-income countries in which the majority of the world’s HIV-positive youth live and the particular settings wherein the rates of HIV-related youth deaths are a major alarm, the absence of screening for mental health disorders, lack of evidence on how to intervene, to prevent or improve mental health problems is an enormous challenge [24].

In Ethiopia, even though HIV services have increased significantly and have been integrated into primary health care, and stated that there is an urgent need for the scale-up of comprehensive mental health services, limited access to these services remains a main challenge to efficiently combat mental health concerns of YLWH [25]. Depression and its related diseases, such as HIV/AIDS, are among the core contributors to compromising the productivity and quality of life of youth. However, the shortage of evidence on mental health disorders such as depression in this age group and inadequate access to mental health services remain the main challenge to successfully struggling with mental health issues of youth, regardless of their high percentage within the general population of Ethiopia [26]. The national prevalence of depression among YLWH in Ethiopia is merely unknown. However, studies in different parts of Ethiopia showed that depression among YLWH ranges from 26.2% − 35.5% [27–30].

Studies so far showed that factors such as residence, age, sex, substance use, behaviorally acquired HIV, non-adherence to ART, non-disclosure of sero-status, hospitalization history, lack/low social support, presence of opportunistic infections, and HIV-related stigma were associated with depression in YLWH [15,21–23,31–35]. Early detection and management of depression are critical to physical health as well as preventing HIV disease progression and transmission. Depression, unfortunately, appears to be undiagnosed in YLWH as health professionals give attention to other medical complaints and also see it as a normal reaction [36].

There is a scarcity of strong evidence on depression and associated factors among YLWH it is crucial to identify the site-specific, varied range of evidence to generate substantial information. Moreover, there was no prior rigorous study done on depression and associated factors among YLWH attending ART clinics in the study zones, where diverse populations with agrarian, pastoralist, and semi-pastoralist lifestyles are present. Previous studies have not assessed the effect of regimen type on depression among HIV-positive youths, but the current study identified initiation on a non-dolutegravir-based regimen as a risk factor. Thus, this study aimed to assess the proportion of depression and associated factors among HIV-positive youth attending ART clinics at public hospitals in Gamo and Ari Zones, South Ethiopia.

## Methods and materials

### Study design, period, and area

A facility-based cross-sectional study was conducted from March 1, 2024, to April 30, 2024, at public hospitals providing HIV/AIDS care and treatment services in Gamo and Ari Zones, South Ethiopia. In the Gamo Zone, there is one General (Arba Minch General Hospital) and five primary hospitals (Kamba Primary Hospital, Geresse Primary Hospital, Dil Fana Primary Hospital, Chencha Primary Hospital, and Selamber Primary Hospitals) currently providing HIV/AIDS care and treatment. Arba Minch town is one of the capital cities of South Ethiopia’s regional state and Gamo Zone, located at a distance of 435 Kilometers (KM) in the south direction of Addis Ababa, the capital city of Ethiopia. In Ari Zone, there is one General (Jinka General Hospital) and one primary hospital (Gazer Primary Hospital) currently providing HIV/AIDS care and treatment. Jinka town is one of the capital cities of South Ethiopia’s regional state and Ari Zone, located at a distance of 585 KM in the south direction of Addis Ababa, the capital city of Ethiopia. This study includes all government general and primary hospitals in the Gamo and Ari Zones. Currently, the total number of HIV-positive youth attending ART clinics in those public hospitals is 737.

### Population

All HIV-positive youth (15–24 years old) attending ART clinics at public hospitals in Gamo and Ari Zones, South Ethiopia, were the source population. Randomly selected HIV-positive youth (15–24 years old) attending ART clinics at public hospitals in Gamo and Ari Zones, South Ethiopia, during the study period, and who fulfilled the eligibility criteria were the study population. All HIV-positive youth (15–24 years old) who attended ART clinics at public hospitals in Gamo and Ari Zones, South Ethiopia, during the study period and who had at least one previous visit to ART clinics were included. Three youth who were seriously ill and unable to attend an interview because of their serious illness were excluded from the study.

### Sample size and sampling procedure

**Fig 1:**
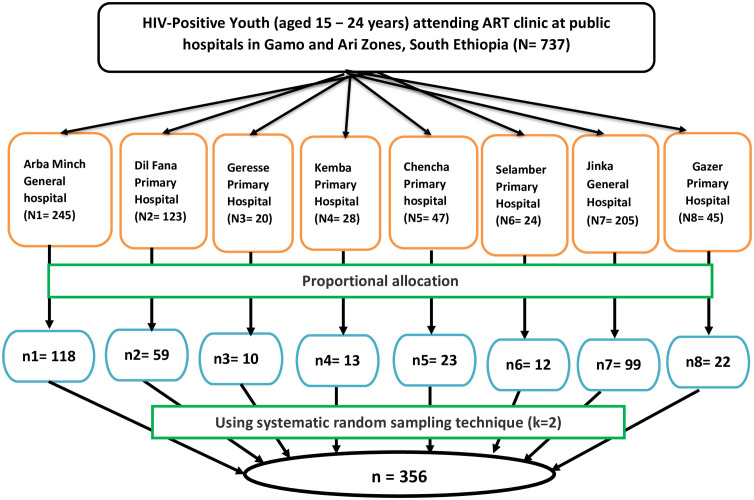
Schematic presentation of the sampling procedure for selecting participants for a study assessing depression and associated factors among HIV-positive youth attending ART clinics at public hospitals in Gamo and Ari Zones, South Ethiopia, 2024.

The sample size was determined using a single population proportion formula by considering the following assumptions: 95% level of confidence, 30.2% proportion of depression among HIV-positive youth from a study conducted in Jimma, Ethiopia [27], and 5% margin of error, and it was 324. After considering the 10% non-response rate, the sample size was 356. A systematic random sampling technique was used to select study participants. All public hospitals in Gamo (Arba Minch General, Dil Fana, Kemba, Geresse, Chencha, and Selamber primary hospitals), and Ari Zone (Jinka General and Gazer primary hospitals) were included in the study. The sample size was allocated proportionally to study facilities. The study participants (HIV-positive youth) were recruited through determining the sampling interval; K [N/n, where N is the total number of HIV-positive youth who attend ART clinics (N = 737) and n is the final sample size determined (n = 356)], which is approximately two. The first participant was selected using the lottery method from the first two patients coming for follow-up. Until the sample size is achieved (n = 356), subjects were selected every two intervals from HIV-positive youth attending follow-up services at the ART clinics during the study period (**Fig 1**).

### Data collection methods and tools

Data was collected using a pretested structured questionnaire to capture information on socio-demographic and economic characteristics, caregiver-related factors, stressor-related factors, behavioral-related factors, psycho-social-related factors, and clinical-related factors. Depression was measured using the PHQ-9 tool. Data was collected using ten senior Bachelor’s degree holders’ nurses working at the ART clinic, and eight health officers and psychiatry nurses had supervised the overall data collection process. Data was collected using a face-to-face interview. Medical records of HIV-positive youth were reviewed using a checklist to extract data on HIV-related variables.

**Depression:** A structured Patient Health Questionnaire-9 (PHQ-9) was used to measure depression among HIV-positive youth. PHQ-9 is a nine-item tool that is directly based on the nine diagnostic criteria for major depressive disorder in the Diagnostic and Statistical Manual Fourth Edition (DSM-IV). For each of the nine items, there is a value set from 0–3, with a total range of 0–27 response scale. The values are: (0 = not at all, 1 = several days, 2 = more than half of the days, 3 = nearly every day). By adding these values, the status of the study participants was determined as depressed if the PHQ-9 score was ≥ 10 [33,37]. PHQ-9 has acceptable reliability with a Cronbach’s-alpha of 0.89, a sensitivity of 88%, and a specificity of 88% for depression diagnosis [38]. Furthermore, PHQ-9 is a reliable and validated tool in Ethiopian patients living with HIV/AIDS and showed good internal and test-retest reliability with Cronbach’s alpha of 0.85 and an intra-class correlation coefficient of 0.92 [39]. Concerning the PHQ-9 tool to diagnose depression, a cut-off score ≥10 can be used regardless of age category (for sub-groups) with a sensitivity of 0.88 and a specificity of 0.85 [40].

**Social support:** The 3-item Oslo‐3 Social Support Scale (OSS‐3) was used to measure youth social support. The tool comprises valid values ranging from 3 to 14. A score was categorized as poor = 3–8, moderate = 9–11, and strong = 12–14. The tool has a Cronbach’s alpha of 0.88 [41].

**Stigma:** Stigma was measured by the 8-item short version of the HIV stigma scale, consisting of a 4-point Likert scale ranging from (strongly disagree, disagree, agree, and strongly agree). The tool consists of questions concerning disclosure status, negative self-image, and public attitudes. The cut-off points above the mean score (18) were considered as the presence of stigma [28]. The tool reliability indicated Cronbach-alpha of 0.81 for internal consistency [42].

**Wealth index:** The wealth index was measured by a simplified and updated Ethiopian wealth index equity tool. The tool contains 15 simplified household asset questions available from www.equitytool.org. The tool has an 84.2% agreement and 0.755 kappa statistics with the full Ethiopian Demographic Health Survey (EDHS, 2016) wealth index measurement tool. Using principal component analysis (PCA), the wealth index of the household was classified into five quintiles [43].

**Physical activity:** Physical activity was assessed using two questions from a physical activity assessment tool. Each question was independently rated, and the sum of the two questions was used to determine the sufficiency of physical activity. Accordingly, those patients who scored ≥4 were categorized as sufficiently active or otherwise insufficiently active [44].

### Study variables

Depression status among HIV-positive youth (Yes or No) was the dependent variable. The independent variables include; socio-demographic variables: age, sex, educational level, residence, wealth index, and occupation; stressors/past-traumatic events variables: education discontinuation due to HIV illness, history of hospital admission, disclosure status, failed school terms, and parental death; behavioral variables: lifetime substance use, current substance use (alcohol, khat, cigarette), and physical activity level; psycho-social support and caregiver variables: HIV-related stigma, social support, living companion, presence of the primary caregiver, primary caregiver types, and change in the primary caregiver; and clinical variables: baseline CD4 count, baseline viral load, current WHO clinical stage, presence of opportunistic infections, ARV side effect, regimen type, history of TB treatment, and years since diagnosis with HIV.

### Operational definitions

**Depressed:** Based on the Patient Health Questionnaire-9 (PHQ-9) score, HIV-positive youth whose score was≥10 were categorized as depressed [27].

**HIV-positive youth:** People between 15 and 24 years of age and who are currently HIV-positive [27].

**Poor social support:** Score ranging from 3−8 based on the 3-item Oslo-3 Social Support Scale (OSS-3). Intermediate support: score ranging from 9−11 based on the 3-item Oslo-3 Social Support Scale (OSS-3). Strong support: a score ranging from 12−14 based on the 3-item Oslo-3 Social Support Scale (OSS-3) [41].

**Substance use:** HIV-positive youth who used either alcohol, shisha, cigarettes, or Khat ever in their life.

**Stigmatized:** Score greater than the mean as measured by the 8-item short version of the HIV stigma scale. Not stigmatized: score less than the mean as measured by the 8-item short version of the HIV stigma scale [42].

**Physical activity:** Patients who scored the two-question physical activity assessment tool ≥4 were categorized as sufficiently active or otherwise insufficiently active [44].

### Data quality management

Data quality was ensured by adopting validated tools by reviewing the literature, giving training to data collectors and supervisors, conducting pretests, and language translation. Accordingly, one-day training was given to the data collectors and supervisors before data collection by investigators on the objective of the study, the method of data collection, and ethical issues. A pretest was done on 5% (36 samples) of the sample size in Wolaita Sodo University Comprehensive Specialized Hospital to check the clarity and consistency of the questionnaires and checklist before the actual data collection. After the pre-test, some amendments, like removal of redundant questions, and clarification of unclear and long questions were made. Discussion on the result of the pre-test and relevant amendments was made as required. Questionnaires will primarily be prepared in English and then translated into the Amharic language and back-translated to English to guarantee consistency. Each completed questionnaire was checked for completeness, clarity, and consistency at the site of data collection by the supervisors to take corrective measures. The overall activities were also monitored by investigators.

### Data processing and analysis

Data were checked, coded, cleaned, and entered into Epi-Data version 3.1. And then exported to STATA version 15 for analysis. Descriptive statistics were computed to describe variables in the study. After checking the assumption, the wealth index was computed using principal component analysis (PCA). Binary logistic regression analysis was used to identify factors associated with depression among HIV-positive youth. A crude odds ratio (COR) with a 95% confidence interval (CI) was used to present a bivariable logistic regression analysis. All variables with p-value <0.25 in the bivariable logistic regression analysis were selected as candidate variables for the multivariable logistic regression analysis to control for confounding. A multivariable logistic regression analysis was performed using a backward stepwise likelihood method to identify factors independently associated with depression. The adjusted odds ratio (AOR) along with a 95% confidence interval (CI) was used to determine the strength of the association. A p-value <0.05 was used to declare statistically significant variables in the final model. Multicollinearity between independent variables was checked using variance inflation factors (VIF) values and the mean VIF = 1.4 and maximum VIF = 2.06, showing no threat of multicollinearity. Model fitness was checked using the Hosmer and Lemeshow goodness-of-fit test and found satisfied (Prob > chi2 = 0.7469). Finally, the findings of the study were presented using texts, tables, and figures.

### Ethical consideration

Ethical clearance was obtained from the Institutional Review Board (IRB) of Arba Minch College of Health Sciences with reference number AMCHS/01/10/3310 on February 24, 2024. Support letters were also obtained from the research and community service directorate office. An official permission letter was obtained from the chief executive director (CEO)/medical directors of each study facility and was given to the ART clinic. Written informed consent for youth ≥18 years old/assent for youth <18 years old was obtained from each study participant. Also, parents/caregivers’ permission was obtained for youth <18 years old. Confidentiality of information was assured throughout the study process. Participants found to have depression were linked to the mental health clinic for further evaluation and management.

## Results

### Sociodemographic and economic characteristics of HIV-positive youths

A total of 343 HIV-positive youths were included in the study with a 96.4% response rate. Of the total study respondents, more than half, 183 (53.3%), were females, and 179 (52.2%) were in the age group 15–19 years. Regarding the educational status of the participants, 48 (14.0%) and 153 (44.6%) attained no formal education and secondary and above, respectively. Gamo ethnicity accounts for 146 (42.6%) of the total study respondents, and Orthodox religion is the dominant religion among the HIV-positive youth recruited in the study. Nearly half, 163 (47.5%) of the HIV-positive youth included in the study were students, and three-fourths were urban residents. The current study identified that 161 (46.9%) and 46 (13.4%) HIV-positive youths were living in households with a poor and medium wealth index (**Table 1**).

**Table 1:** Socio-demographic characteristics of HIV-positive youths attending ART clinics at public hospitals in Gamo and Ari Zones, South Ethiopia, 2024 (n = 343).

Variables	Categories	Frequency	Percent
Sex	Male	160	46.7
	Female	183	53.3
Age	15-19 years	179	52.2
	20-24 years	164	47.8
Formal education	No formal education	48	14.0
	1-8 grade	142	41.4
	Secondary and above	153	44.6
Ethnicity	Gamo	146	42.6
	Goffa	53	15.5
	Ari	58	16.9
	Wolaita	44	12.8
	Others*	42	12.2
Religion	Orthodox	173	50.4
	Protestant	154	44.9
	Others**	16	4.7
Occupational status	Unemployed	58	16.9
	Government employee	11	3.2
	Self-employed	111	32.4
	Student	163	47.5
Residence	Urban	259	75.5
	Rural	84	24.5
Wealth index	Poor	161	46.9
	Medium	46	13.4
	Rich	136	39.7

*Konso, Derashe, Mursi, and Hamer; **Catholic, Apostolic, and Adventist

### Psychosocial and stressor-related characteristics of HIV-positive youths

Of the total study participants, 148 (43.2%) received intermediate-level psychosocial support; more than half, 167 (51.3%) were affected by HIV-related stigma, and nearly two-thirds (64.4%) of the youths are living with their families. Regarding primary caregivers, more than two-thirds, 232 (67.6%), of the youths living with HIV have primary caregivers. In the last 12 months before the survey, only 34 (9.9%) youth living with HIV had a hospital admission. The HIV-sero status of more than half, 192 (56.0%), of the study respondents was disclosed to others, and among this, the HIV-sero status of 113 (58.8%) respondents was disclosed to family members other than the primary caregiver. The biological parents of 90 (38.5%) youths living with HIV died, among whom the father’s death accounted for 52 (42.4%) (Table 2).

**Table 2:** Psychosocial and stressor-related characteristics of HIV-positive youths attending ART clinics at public hospitals in Gamo and Ari zones, South Ethiopia, 2024 (n = 343).

Variables	Categories	Frequency	Percent
Psychosocial support	Poor support	133	38.8
	Intermediate support	148	43.2
	Strong support	62	18.0
Stigma	Yes	167	51.3
	No	176	48.7
Living condition	With family	221	64.4
	With relatives	48	14.0
	Alone	74	21.6
Have a primary caregiver	Yes	232	67.6
	No	111	32.4
Type of primary caregiver (n = 243)	Both parents	96	39.5
	Mother only	71	29.2
	Father only	28	11.5
	Siblings	29	11.9
	Others	19	7.8
Primary caregiver changed since being HIV positive (n = 243)	Yes	49	20.2
	No	194	79.8
Hospital admission in the last 12 months	Yes	34	9.9
	No	309	90.1
Disclosure	Yes	192	56.0
	No	151	44.0
To whom you disclose (n = 192)	Primary caregiver	75	39.1
	Other family members	113	58.8
	Everyone	4	2.1
Ever failed in school terms	Yes	106	30.9
	No	237	69.1
Ever discontinue school terms	Yes	53	15.4
	No	290	84.6
Death of biological parents	Yes	90	38.5
	No	211	61.5
Who died among biological parents (n = 132)	Both mother and father	43	32.6
	Mother only	33	25.0
	Father only	56	42.4
Teasing for appearance after HIV diagnosis	Yes	102	29.7
	No	241	70.3
Teasing for appearance after ART drug	Yes	93	27.1
	No	250	72.9

### Behavioral and clinical-related characteristics of HIV-positive youths

About two-thirds, 222 (64.7%), of the study participants use substances including alcohol, cigarettes, Khat, and shisha. In the past 3 three months, 27 (7.9%), 3 (0.9%), 5 (1.4%), and 2 (0.6%) of the youths living with HIV used alcohol, cigarettes, Khat, and shisha once or twice, respectively. Regarding physical exercise, the majority, 289 (84.3%), of the HIV-positive youths were doing sufficient exercise. Regarding opportunistic infection, only 21 (6.1%) and 60(17.5%) of the participants had a history of TB and OI other than TB infection, respectively. Ninety percent of the study participants are on the first-line antiretroviral regimen, among which the majority, 302 (88.0%), are Dolutegravir (DTG) based regimen. A majority, 293 (85.4%), of the study respondents lived with HIV for more than 12 months (Table 3).

**Table 3:** Behavioral and clinical-related characteristics of youths attending antiretroviral therapy clinics at public hospitals in Gamo and Ari zones, South Ethiopia, 2024 (n = 343).

Variables	Categories	Frequency	Percent
Substance use	Yes	222	64.7
	No	121	35.3
Alcohol use	Yes	212	61.8
	No	131	38.2
Frequency of alcohol use in the past 3 months	Never	263	76.7
	Once or twice	27	7.9
	1 to 3 times per month	42	12.2
	1 to 4 times per week	11	3.2
Cigarette smoking	Yes	16	4.7
	No	327	95.3
Frequency of cigarette smoking in the past 3 months	Never	333	97.1
	Once or twice	3	0.9
	1 to 3 times per month	5	1.4
	1 to 4 times per week	2	0.6
Khat chew	Yes	40	11.7
	No	303	88.3
Frequency of Khat chewing in the past 3 months	Never	310	90.4
	Once or twice	5	1.4
	1 to 3 times per month	22	6.4
	1 to 4 times per week	6	1.8
Shisha smoking	Yes	10	2.9
	No	333	97.1
Frequency of smoking shisha in the past 3 months	Never	337	98.3
	Once or twice	2	0.6
	1 to 3 times per month	3	0.9
	1 to 4 times per week	1	0.2
Physical exercise	Sufficiently active	289	84.3
	Insufficiently active	54	15.7
Ever missed the ART drug	Yes	86	25.1
	No	257	74.9
Baseline CD4 count (n = 220)	<200 cell/mm3	27	12.3
	≥200 cell/mm3	193	87.7
Baseline viral load (n = 105)	<1000 copies/ml	95	90.5
	≥1000 copies/ml	10	9.5
WHO clinical staging	I – II	295	86.0
	III – IV	48	14.0
Presence of OI other than TB	Yes	60	17.5
	No	283	82.5
Current ART regimen	First line	311	90.7
	Second	31	9.0
	Third line	1	0.3
DTG-based regimen	Yes	302	88.0
	No	41	12.0
Side effect	Yes	4	1.2
	No	339	98.8
TB infection	Yes	21	6.1
	No	322	93.9
Duration since HIV diagnosis	≤12 months	50	14.6
	>12 months	293	85.4

### Proportion of depression among HIV-positive youths

**Fig 2:**
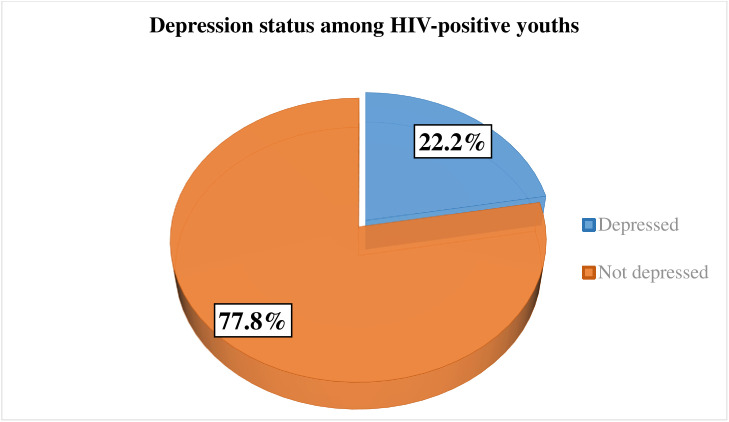
Proportion of depression among HIV-positive youths attending ART clinics at public hospitals in Gamo and Ari zones, South Ethiopia, 2024.

The proportion of depression among HIV-positive youth attending ART clinics at public hospitals in Gamo and Ari zones, South Ethiopia, was found to be 76 (22.2%; 95% CI: 18.1%, 26.9%) (Fig 2).

### Factors associated with depression among HIV-positive youths

In bivariable logistic regression analysis, sex, age, educational status, wealth index, psychosocial support, history of hospital admission, disclosure status, ever-failed school terms, death of the parents, history of substance use, physical exercise, WHO clinical staging, history of TB infection, presence of OI other than TB infection, DTG-based regimen, and duration since HIV-positive are identified to be significantly associated with depression among HIV-positive youths at a p-value of 0.25 and were candidates for multivariable analysis. In multivariable logistic regression, after controlling for confounding variables, psychosocial support, death of the parent, substance use, WHO clinical staging and utilization of a non-Dolutegravir-based regimen showed statistically significant association with depression at p-value <0.05 (Table 4).

**Table 4:** Bivariable and multivariable logistic regression of factors associated with depression among HIV-positive youths attending antiretroviral therapy clinics at public hospitals in Gamo and Ari zones, South Ethiopia, 2024 (n = 343).

Variables	Categories	Depression		COR (95% CI)	P-value	AOR (95% CI)	P-value
		Yes (%)	No (%)				
Sex	Male	49 (30.6)	111 (69.4)	1		1	
	Female	27 (14.8)	156 (85.2)	0.39 (0.23, 0.67)	0.001	0.79 (0.40, 1.54)	0.49
Age	15-19 years	46 (25.7)	133 (74.3)	1		1	
	20-24 years	30 (18.3)	134 (81.7)	0.65 (0.39, 1.09)	0.100	0.88 (0.45, 1.71)	0.70
Wealth index	Poor	51 (31.7)	110 (68.3)	3.74 (1.99, 7.03)	<0.001	2.12 (0.99, 4.53)	0.053
	Medium	10 (21.7)	36 (78.3)	2.24 (0.93, 5.42)	0.073	1.82 (0.65, 5.08)	0.25
	Rich	15 (11.0)	121 (89.0)	1		1	
Psychosocial support	Poor support	53 (39.8)	80 (60.2)	3.90 (1.78, 8.57)	0.001	2.87 (1.18, 6.98)	0.02*
	Intermediate support	14 (9.5)	134 (90.5)	0.62 (0.25, 1.51)	0.288	0.56 (0.21, 1.49)	0.25
	Strong support	9 (14.5)	53 (85.5)	1		1	
Hospital admission	Yes	13 (38.2)	21 (61.8)	2.42 (1.15, 5.09)	0.020	1.30 (0.45, 3.79)	0.63
	No	63 (20.4)	246 (79.6)	1		1	
Ever failed in school terms	Yes	30 (28.3)	76 (71.7)	1.64 (0.96, 2.79)	0.068	1.12 (0.54, 2.33)	0.77
	No	46 (19.4)	191 (80.6)	1		1	
Death of parents	Yes	54 (40.9)	78 (59.1)	5.95 (3.39, 10.43)	<0.001	3.28 (1.73, 6.20)	<0.001
	No	22 (10.4)	189 (89.6)	1		1	
Substance use	Yes	64 (28.8)	158 (71.2)	3.68 (1.90, 7.14)	<0.001	3.44 (1.60, 7.43)	0.002
	No	12 (9.9)	109 (90.1)	1		1	
Physical exercise	Sufficiently active	57 (19.7)	232 (80.3)	1		1	
	Insufficiently active	19 (35.2)	35 (64.8)	2.21 (1.18, 4.15)	0.014	1.72 (0.80, 3.71)	0.17
WHO clinical staging	I – II	49 (16.6)	246 (83.4)	1		1	
	III – IV	27 (56.2)	21 (43.8)	6.45 (3.38, 12.33)	<0.001	4.35 (1.97, 9.61)	<0.001*
Presence of OI other than TB	Yes	29 (48.3)	31 (51.7)	4.70 (2.59, 8.52)	<0.001	1.56 (0.59, 4.12)	0.37
	No	47 (16.6)	236 (83.4)	1		1	
DTG-based regimen	Yes	55 (18.2)	247 (81.8)	1		1	
	No	21 (51.2)	20 (48.8)	4.72 (2.39, 9.29)	<0.001	2.72 (1.15, 6.42)	0.022*
TB infection	Yes	10 (47.6)	11 (52.4)	3.53 (1.44, 8.66)	0.006	0.70 (0.18, 2.78)	0.61
	No	66 (20.5)	256 (79.5)	1		1	
Duration since HIV diagnosis	≤12months	5 (10.0)	45 (90.0)	0.35 (0.13, 0.91)	0.031	0.52 (0.16, 1.74)	0.29
	>12months	71 (24.2)	222 (75.8)	1		1	

*Significant at p-value <0.05.

Youths with poor psychosocial support had more than two-fold times increased odds of being depressed than children with strong psychosocial support (AOR = 2.87; 95%CI: 1.18, 6.98). Death of parents increases the risk of depression by 3.28 times as compared to their complements (AOR = 3.28; 95%CI: 1.73, 6.20). Youth who use substances like alcohol, cigarettes, khat, and shisha had more than three times higher odds of depression than their counterparts (AOR = 3.44, 95%CI: 1.60, 7.43). The risk of being depressed was increased by 4.35 times among youths with advanced WHO clinical staging (stage III/IV) when compared to their counterparts (stage I/II) (AOR = 4.35; 95%CI: 1.97, 9.61). Moreover, youths who are on a non-DTG-based regimen were more likely to be depressed compared to those on a DTG-based regimen (AOR = 2.72; 95%CI: 1.15, 6.42) (Table 4).

## Discussion

This study aimed to assess the proportion of depression and associated factors among HIV-positive youths. Accordingly, the proportion of depression was determined to be 22.2% (95% CI: 18.1%, 26.9%). Moreover, poor psychosocial support, death of parents, substance use, advanced WHO clinical stage, and being on a DTG-based regimen were the factors identified to be significantly associated with depression among HIV-positive youths.

The findings of this study was in line with that of cross-sectional studies done in the USA [45,46] among HIV-positive youths. It is also supported by a prospective study done in England [47], a mixed study design done in Zambia [35], a cross-sectional study in Rwanda [48], and Kenya [49] that reported a proportion of depression of 16%, 25.3%, 26%, and 28.8%, respectively. Moreover, the findings of the current study was also consistent with evidence from studies previously conducted in different parts of Ethiopia, like Dessie [29] and Jimma [27], among HIV-positive youths, revealed that the proportion of depression was 26.2% and 30.2%, respectively.

Evidence from the current study reported that the overall proportion of depression was nearly one in four. This is smaller than the finding from studies conducted in Philadelphia [16], five USA countries (New York City, Chicago, Miami, Los Angeles, and New Orleans) [50], USA [51], Jamaica [15], and South Africa [52] which reported 52%, 43.3%, 35%, 62%, and 33.8%, respectively. It is also smaller than the findings from a previous study done in Addis Ababa, Ethiopia [30] and another study done in nine hospitals, in Addis Ababa, Ethiopia [28], which reported that 31.7% and 35.5%, respectively. On the other hand, the overall proportion of depression among HIV-positive youths depicted by the current study was slightly higher than that of the cross-sectional studies conducted in Ukraine [53], a cross-sectional study done in Thai [37], and Philadelphia [7], which reported a proportion of depression of 13%, 11%, and 12.2%, respectively. The possible explanation for the discrepancy might be the dissimilarity in the source population, as studies done in Ukraine [53] included youths aged 13–25 years, that of Philadelphia [7] included individuals aged 18–30 years old, and that of South Africa [52] included those aged 13–24 years. The variability in tools to measure the proportion of depression among studies might also be responsible for the discrepancy. The studies done in the United States of America (USA) [50], Zambia [35], and Rwanda [48] used the Center of Epidemiological Studies (CES-II) to determine the proportion of depression. Whereas studies done in Jamaica [15], Ukraine [53], and Addis Ababa, Ethiopia [28] use the Depression Anxiety Stress Scale (DASS-21), Hospital Anxiety and Depression Scale (HADS-14), and Beck Depression Inventory (BDI) tools, respectively. Moreover, the discrepancy also might be explained by the variability in sample size, study design, study settings, and sociodemographic characteristics of the study participants.

The current study reported that youths with poor psychosocial support had more than two-fold increased odds of being depressed than youths with strong psychosocial support. This finding is consistent with the evidence from the study conducted in Dessie [29] and Addis Ababa, Ethiopia [28] showed that the odds of depression were higher among youth with low psychosocial support. It is also supported by the findings from a cross-sectional study done in Addis Ababa, Ethiopia [54], which revealed that HIV-positive individuals with poor social support had two times higher risk of being depressed than their counterparts. The possible explanation might be that youths living with HIV without a close family/caregiver to discuss their problem can’t get the appropriate care they need at any time and may have more frequent depressive symptoms than their counterpart [55]. It could also be because of different factors such as debilitation, educational disability, isolation, and food insecurity. Poor psychosocial support might result in suboptimal drug adherence, and because of this, suboptimal drug adherence results in immune suppression, which eventually leads to depression [56].

The other factor associated with depression among youths living with HIV was parental death. The death of parents increases the risk of depression by more than three times in youth with no parental death. This might be due to the reason that youth are living with their parents and are financially dependent on them. Hence, family death has a critical influence on the youth’s psychological well-being. Losing a parent can lead to feelings of grief, sadness, loneliness, and anxiety, which can contribute to the development of depression [57,58]. The emotional impact of losing a parent can also be compounded by the stigma and discrimination that HIV-positive youths may face, as well as concerns about their health and well-being.

Youth who use substances such as alcohol, cigarettes, khat, and shisha had more than three times higher odds of depression than their counterparts. This is supported by studies conducted in Jamaica [15], Thai [37], and South Africa [52] among HIV-positive youth, stating substance users had more than three times the risk of being depressed. This might be because substance use can lead to a neurotransmitter imbalance in the human brain, which results in mood disturbance and depression. Among HIV-positives, substance use can expose them to drug non-adherence, which may fasten disease progression and worsen the health outcome, and this will contribute to the increased feeling of depression and hopelessness [59].

The risk of being depressed was increased by 4.35 times among youths with advanced WHO clinical staging (stage III/IV) when compared to their counterparts. This evidence is supported by the findings from a cross-sectional study done in Addis Ababa, Ethiopia [54] which showed that HIV-positive individuals with advanced WHO clinical staging (stage III) had nearly three-fold times higher odds of depression than their counterparts. Likewise, the likelihood of depression was reduced by 84% among HIV-positive women with stage I when compared to those with advanced WHO clinical staging [60]. The plausible explanation behind this is that the risk of depression was seen to increase with the HIV WHO clinical staging of the disease progression. Averting disease progression is crucial by promoting HIV treatment and care, mainly ART, which highly contributes to preventing depression [61].

Moreover, youths who are on a non-DTG-based regimen were more likely to be depressed compared to those on a DTG-based regimen. Twelve percent of the youths living with HIV are on a non-DTG-based regimen in this study. The possible explanation is that Efavirenz, Zidovudine, Abacavir, and Nevirapine-based regimens have been associated with neuropsychiatric side effects, which might lead to depression [62].

### Strengths and limitations of the study

As a limitation, it might be difﬁcult to establish a temporal relationship because of the nature of the study design. Even if a due emphasis was given to remembering past events, there might be some recall bias among the study participants. For substance use-related questions, participants might have given socially acceptable responses. Emphasis on intensive training, pretesting, and active field data collection supervision to minimize bias was given.

## Conclusion

Depression among HIV-positive youths is a significant public health problem in the study setting. Poor psychosocial support, death of parents, substance use, advanced WHO clinical staging (stage III/IV), and initiation on a non-DTG-based regimen were the factors associated with depression among HIV-positive youths. Special attention should be given to youths who are in poor psychosocial support, substance use, orphaned, and those with poor baseline clinical characteristics. Extensive parent/caregiver involvement in the care of the youths to improve psychosocial support and tackle harmful substance use is highly encouraged. Special consideration should be given to youths who are taking on old regimens they started on at baseline. Moreover, early identification, linkage, and treatment of youths with depressive symptoms during routine HIV care are essential to avert depression. A further prospective study is necessary to delineate factors and to establish the direction of associations.

## Supporting information

S1 Dataset The dataset supports the findings of the study. (XLS)

## Abbreviation:

AIDS: acquired immuno-deficiency syndrome
AOR: adjusted odds ratio
ART: anti-retroviral therapy
ARV: antiretroviral
BDI: beck depression inventory
CEO: chief executive officer
CD4: cluster of differentiation 4
CES-II: center of epidemiological studies II
CI: confidence interval
COR: crude odds ratio
DASS-21: depression anxiety stress scale 21
DSM-IV: diagnostic and statistical manual fourth edition
DTG: dolutegravir
EDHS: ethiopian demographic health survey
HIV: human immuno-deficiency virus
IRB: institution review board
NCD: non-communicable disease
OI: opportunistic infections
OSS-3: oslo-social support scale-3
PCA: principal component analysis
PHQ-9: patient health questionnaire-9
PI: principal investigator
PLWH: patient living with HIV/AIDS
SSA: sub-Saharan Africa
TB: tuberculosis
USA: United States of America
VIF: variance inflation factor
WHO: World Health Organization
YLD: years lost due to disability
YLWH: youth living with HIV/AIDS
